# An Improved Deep Learning Model: S-TextBLCNN for Traditional Chinese Medicine Formula Classification

**DOI:** 10.3389/fgene.2021.807825

**Published:** 2021-12-22

**Authors:** Ning Cheng, Yue Chen, Wanqing Gao, Jiajun Liu, Qunfu Huang, Cheng Yan, Xindi Huang, Changsong Ding

**Affiliations:** ^1^ School of Informatics, Hunan University of Chinese Medicine, Changsha, China; ^2^ Big Data Analysis Laboratory of Traditional Chinese Medicine, Hunan University of Chinese Medicine, Changsha, China

**Keywords:** S-TextBLCNN model, deep learning, formula classification, formula-vector, data imbalance

## Abstract

**Purpose:** This study proposes an S-TextBLCNN model for the efficacy of traditional Chinese medicine (TCM) formula classification. This model uses deep learning to analyze the relationship between herb efficacy and formula efficacy, which is helpful in further exploring the internal rules of formula combination.

**Methods:** First, for the TCM herbs extracted from *Chinese Pharmacopoeia*, natural language processing (NLP) is used to learn and realize the quantitative expression of different TCM herbs. Three features of herb name, herb properties, and herb efficacy are selected to encode herbs and to construct formula-vector and herb-vector. Then, based on 2,664 formulae for stroke collected in TCM literature and 19 formula efficacy categories extracted from *Yifang Jijie*, an improved deep learning model TextBLCNN consists of a bidirectional long short-term memory (Bi-LSTM) neural network and a convolutional neural network (CNN) is proposed. Based on 19 formula efficacy categories, binary classifiers are established to classify the TCM formulae. Finally, aiming at the imbalance problem of formula data, the over-sampling method SMOTE is used to solve it and the S-TextBLCNN model is proposed.

**Results:** The formula-vector composed of herb efficacy has the best effect on the classification model, so it can be inferred that there is a strong relationship between herb efficacy and formula efficacy. The TextBLCNN model has an accuracy of 0.858 and an F_1_-score of 0.762, both higher than the logistic regression (acc = 0.561, F_1_-score = 0.567), SVM (acc = 0.703, F_1_-score = 0.591), LSTM (acc = 0.723, F_1_-score = 0.621), and TextCNN (acc = 0.745, F_1_-score = 0.644) models. In addition, the over-sampling method SMOTE is used in our model to tackle data imbalance, and the F_1_-score is greatly improved by an average of 47.1% in 19 models.

**Conclusion:** The combination of formula feature representation and the S-TextBLCNN model improve the accuracy in formula efficacy classification. It provides a new research idea for the study of TCM formula compatibility.

## 1 Introduction

The Chinese herbal formula is the connection of traditional Chinese medicine (TCM) basic theory and clinic, and it is also the link between syndrome differentiation and treatment of TCM. TCM treatment relies on formula efficacy to regulate body balance and to treat diseases, and the efficacy is the collective effect of combining a set of herbs. (Fu et al., 2012). Clarifying the modern scientific connotation of the implied relationships between formula compatibility and efficacy systematically is a pressing problem in modern formula research. It is also a major direction for the inheritance and innovation of TCM. A large number of clinical medical records accumulated by ancient physicians, especially refined formulae, provide the basis for the analysis of formulae and efficacy. However, TCM theory is empirical and lacks objective standards. The relationships among formulae, herbs, and efficacy are complicated. In addition, the text grammar and expressions in ancient Chinese medicine books are obscure and difficult to understand in ancient language format, which is different from modern Chinese (Li and Yang, 2017), and the text structure of TCM formulae is mostly unstructured or semi-structured. Therefore, it is hard to translate it into a computer language. Moreover, in the medical field, clinical records of common diseases are usually much more than rare diseases with few recorded cases, resulting in an imbalance of formula data with different efficacies. Data imbalance often impairs model prediction (Indraswari et al., 2019; Yeh et al., 2020). Therefore, these issues have caused many difficulties and challenges in TCM formulae research.

As a carrier of TCM theory, diagnosis and treatment, formulae in the form of text are abstract and vague in expression. Therefore, quantification of TCM formulae efficacies is an urgent issue in analyzing formula compatibility. It could be solved by natural language processing (NLP). NLP, which can convert nonstandard text into structured data, now is widely used to process electronic medical records (Dharmage et al., 2019) and predict protein sequences (Ofer et al., 2021). In particular, Word2Vec is a technology of language modeling in the NLP field. In order to represent words’ semantic information, Word2Vec maps vocabulary words to fixed-length vectors based on word distribution hypotheses. At present, Word2Vec is widely used not only in dialogue act recognition (Cerisara et al., 2018), topic classification (Daouadi et al., 2021; Mohamed et al., 2021), and sentiment analysis (Bao et al., 2020; Muhammad et al., 2021) but also in the field of medical science (Ji et al., 2020; Richard and Reddy, 2020). However, studies have shown that simply using the NLP method is not entirely feasible because there are certain differences between natural language and TCM formulae (Li and Yang, 2017). We consider that each herb encoded by the name itself cannot describe herb features well, so the herb attributes including the herb name, four characters, five tastes, channel tropism, toxicity, and efficacy could be used as features. Then, the herb-vector is constructed through training on a language model to represent features and to establish the relationships among herbs. According to the herb-vector, we construct the formula-vector, which helps to further analyze the relationship between herb and formula efficacy.

In recent years, using artificial intelligence to study formula efficacy and formula combination has become a current research hotspot. Machine learning and deep learning have been used in TCM research due to their non-linear fitting characteristic (Kowsari et al., 2019). Machine learning is increasingly being used to overcome this basic problem of representation, prediction, and treatment selection in the branch of medicine (Schultebraucks et al., 2019). The advantage of machine learning is that it analyzes various data types and integrates them into the research in disease risks, diagnosis, prognosis, and appropriate treatment (Kee et al., 2019), such as disease risk prediction (Poplin et al., 2018), tongue diagnosis (Meng et al., 2017; Dai and Wang, 2018), medication rule analysis (You et al., 2019), and prediction of the risk of medicine-induced injury (Saini et al., 2018). Deep Learning (DL) uses a multilayer neural network structure to decompose complex mappings into a series of nested simple mappings and extracts from local features to overall features layer by layer to solve complex problems. Currently, DL has solved many practical problems in the field of TCM such as case classification (Song et al., 2019), Chinese herbal medicine identification (Weng et al., 2017), disease risk prediction (Poplin et al., 2018), syndrome differentiation (Hu et al., 2018), and herbal analysis (Zitnik et al., 2018; Yeh et al., 2020). Although DL has been widely used in many application fields, TCM formula efficacy has received much less attention. DL has a good non-linear fitting ability, and it could be used to understand the rules of syndrome differentiation and herb combination. A study has pointed out that RNN and word2vec embedding are the most popular methods in clinical NLP tasks (Wu et al., 2019). We believe that DL combined with formula-vector will have good performance in predicting TCM formula efficacy.

One of the main problems in the formula efficacy classification is data imbalance. Generally, formulae used to treat common diseases account for a large proportion of samples. For uncommon diseases, only a few samples can be obtained from classic ancient books. Therefore, the data imbalance problem should be paid attention to during the model building process. In the case of training formula efficacy prediction models on unbalanced data sets, mainstream machine learning algorithms are trained to predict samples of the majority group in the background that the positive samples being the minority, and the negative samples being the majority. In this way, although a higher accuracy rate can be obtained, it will end up with extremely low recall rate. Therefore, the predictive model cannot classify the positive samples correctly. At present, the over-sampling technology and under-sampling technology are commonly used in solving the problem of data imbalance (de Morais and Vasconcelos, 2019; Makkar and Kumar, 2019).

**FIGURE 1:**
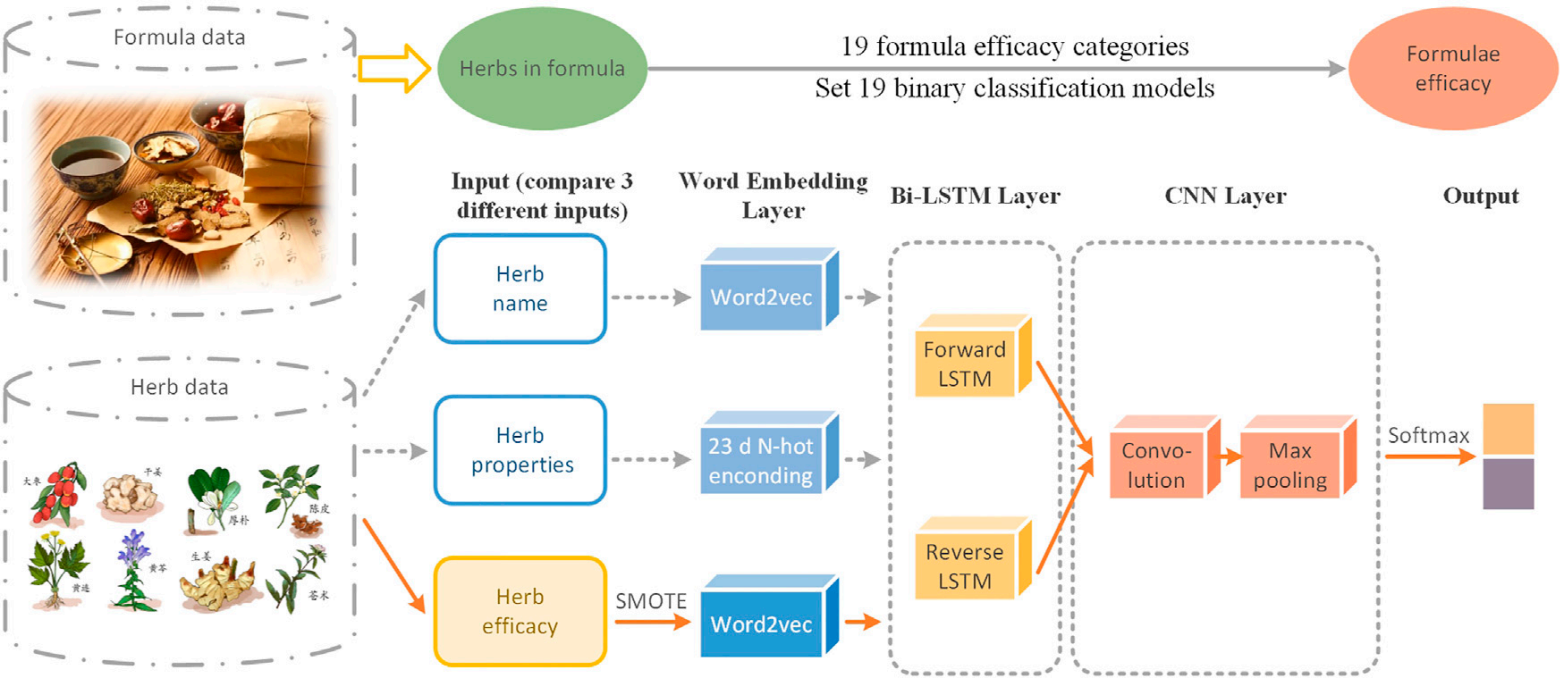
Workflow of S-TextBLCNN.

Our research is at the intersection of TCM and DL classification. Both of them are mature fields with a long history and abundant research studies. We believe that applying machine learning, DL, and other technologies to analyze a large number of formula samples can discover the inner rules of formulae compatibility. A S-TextBLCNN model is proposed for formula efficacy classification (Figure 1). The main work of this model is to use Bi-LSTM and CNN for extracting the features of formulae to predict formula efficacy. It also uses SMOTE to solve the problem of data imbalance. As far as we know, this is the first DL framework for the task of predicting formula efficacy. Our finding provides a new way to study the efficacy of TCM formula classification.

## 2 Materials and Methods

### 2.1 Data Sets

We select 2,664 formulae for stroke extracted from ancient to modern books, which contain TCM classics Chinese medicine journals from the 1980s to the 2010s and various TCM classics, such as *Treatise on Exogenous Febrile Diseases* (《伤寒论》) and *Synopsis of Prescriptions of the Golden Chamber* (《金匮要略》). We get a relatively comprehensive collection of ancient and modern general formulae for the treatment of stroke, including for the treatment of transient cerebral ischemia, cerebral hemorrhage, cerebral infarction, stroke complications, and stroke sequelae. The formula data mainly include formula name, ingredients, and efficacy.

The classification methods of formulae develop with the development of Chinese herbal formulae. As early as *The Inner Canon of Huangdi* (《黄帝内经》) put forward the theory of “Seven Formulae” depending on the composition of formulae, *Shanghan Mingli Lun* (《伤寒明理论》) clearly divided formulae into “big, small, slow, urgent, odd, even, and complex”. Zangqi Chen put forward “promulgating, stimulating, replenishing, venting, light, heavy, slippery, astringent, dry, and damp” in *Gleaning Herb* (《本草拾遗》), which is called “Ten Ji” by later generations. In the Qing Dynasty, Jingyue Zhang proposes “Eight Zhen” in *Jingyue Quanshu*, namely, “complement, harmony, attack, disperse, cold, heat solid, and cause”. With the development of the society and the improvement of understanding levels, the formula classification gradually tends to be perfect and reasonable. According to the efficacy and disease syndrome, Wang Ang’s *Yifang Jijie* (《医方集解》) in the Qing Dynasty divided the formulae into 21 categories. Although there are many ways to classify formulae, the most commonly used method at present is a comprehensive classification combining disease syndrome and corresponding formula functions. Therefore, we classify the formulae based on *Yifang Jijie* referenced as a scientific and systematic comprehensive classification method. With a total of 21 categories, the formula we collected on treatment of stroke does not include the functions of deworming and multiparity, so the formula efficacy based on our data is divided into 19 categories as follows moistening dryness (治燥), invigoration (补益), regulating blood (理血), dispelling pathogenic (祛风), removing phlegm (祛痰), carbuncle (痈疡), eliminating dampness (祛湿), regulating Qi-flowing (理气), warming (温里), reconciliation (和解), clearing heat (清热), emergency (救急), tranquillization (安神), improving eyesight (明目), purgation (泻下), resuscitating (开窍), resolving food (消食), astringing (固涩), and relieve exterior syndrome (解表). We sort out the original formula efficacy and divided formulae into these 19 categories. Part of the formula data is shown in Table 1. In the following research, we will separately establish a binary classification model for each category of formula efficacy. The positive samples of each efficacy classification model are formulae with this efficacy, and the negative samples are the formulae without this efficacy in a total of 2,664 samples.

**TABLE 1:** Partial formula data.

Formula name	Formula ingredients	Original formula efficacy	Formula efficacy after preprocessing
BYHWT (补阳还五汤)	Huang Qi (黄芪), Dang Gui (当归), Chi Shao (赤芍), Di Long (地龙), Chuang Xiong (川芎), Hong Hua (红花), Tao Ren(桃仁)	promoting blood, free collateral vessels, invigorating Qi	regulating blood, invigoration
JYT (解语汤)	Qiang Huo (羌活), Fang Feng (防风), Tian Ma (天麻), Rou Gui (肉桂), Chuang Xiong (川芎), Nan Xing (南星), Dang Gui (当归), Ren Shen (人参), Gan Cao(甘草)	dispelling pathogenic, removing phlegm, free collateral vessels	dispelling pathogenic, removing phlegm, regulating blood
TMGTY (天麻钩藤饮)	Tian Ma (天麻), Chuan Niu Xi (川牛膝), Gou Teng (钩藤), Jue Ming Zi (决明子), Hei Shan Zhi (黑山栀), Du Zhong (杜仲), Huang Cen(黄芩), Yi Mu Cao(益母草), Sang Ji Sheng (桑寄生), Shou Wu Teng (首乌藤), Fu Shen (茯神)	tranquillizing liver yang, calming endogenous wind, clear heat, promoting blood, benefiting liver Qi and invigorating kidney Qi	dispelling pathogenic, clearing heat, regulating blood, invigoration
TYT (通幽汤)	Zhi Gan Cao(炙甘草), Hong Hua (红花), Sheng Di Huang (生地黄), Shu Di Huang (熟地黄), Sheng Ma (升麻), Tao Ren(桃仁), Dang Gui (当归)	nourishing blood, promoting blood, moistening dryness	moistening dryness, invigoration, regulating blood
XCQT (小承气汤)	Da Huang (大黄), Zhi Shi (枳实), Hou Pu(厚朴)	relax bowels, direct Qi downward to relieve hiccup	purgation, regulating Qi-flowing

According to the *Chinese Pharmacopoeia* (《中国药典》), we collect the information of 1,054 TCM herbs, including herb name, four characters (cold, hot, warm, cool, and neutral), five tastes (sour, bitter, sweet, pungent, and salty), channel tropism (lung, pericardium, heart, large intestine, triple energizers, small intestine, stomach, gallbladder, bladder, spleen, liver, and kidney), toxicity, and efficacy. Part of the herb data is shown in Table 2. The combination of four characters, five tastes, channel tropism, and toxicity of TCM herbs is called herb properties. In subsequent experiments, herb name, herb properties, and herb efficacy will be separately used as the features of the formula classification study.

**TABLE 2:** Partial herb data.

Herb name	Four characters	Five tastes	Channel tropism	Toxicity	Herb efficacy
Huang Qi (黄芪)	Warm	Sweet	Lung, spleen	No	tonify Qi, secure exterior, diuresis, expel toxin, expel pus, promoting tissue regeneration
Dang Gui (当归)	Warm	Sweet, spicy	Heart, spleen, liver	No	nourishing blood, promoting blood, regulate menstruation, relieve pain, relax the bowels
Hei Shan Zhi (黑山栀)	Cold	Bitter	Lung, heart, triple energizers	No	clear heat, purge fire, drain dampness, cooling blood, detoxify, disperse swelling, relieve pain
Fu Zi (附子)	Hot	Sweet, spicy	Heart, spleen, kidney	Yes	restoring Yang, save from collapse, tonify fire, assist yang, dissipate cold, relieve pain
Gou Qi Zi (枸杞)	Neutral	Sweet	Liver, kidney	No	tonify the liver and kidney, invigorating Qi, improving vision

### 2.2 Formula-Vector

In this section, we describe three different methods for setting formula-vector. The formula-vector is used as input for the word embedding layer.

One-hot and Word2ve2c are both used in the word embedding layer. One-hot representation is a commonly used method to use a long vector to represent each word separately. The dimensions of a vector represent the size of the vocabulary. Most elements in each vector are ‘0’, and only one of them is a “1”. The position of “1” indicates the position in the vocabulary of the word represented by this vector. Word2vec (Mikolov et al., 2013), as an NLP modeling and feature learning technology, maps words or phrases in the vocabulary into fixed-length real-number vectors to represent the word semantics.

**FIGURE 2:**
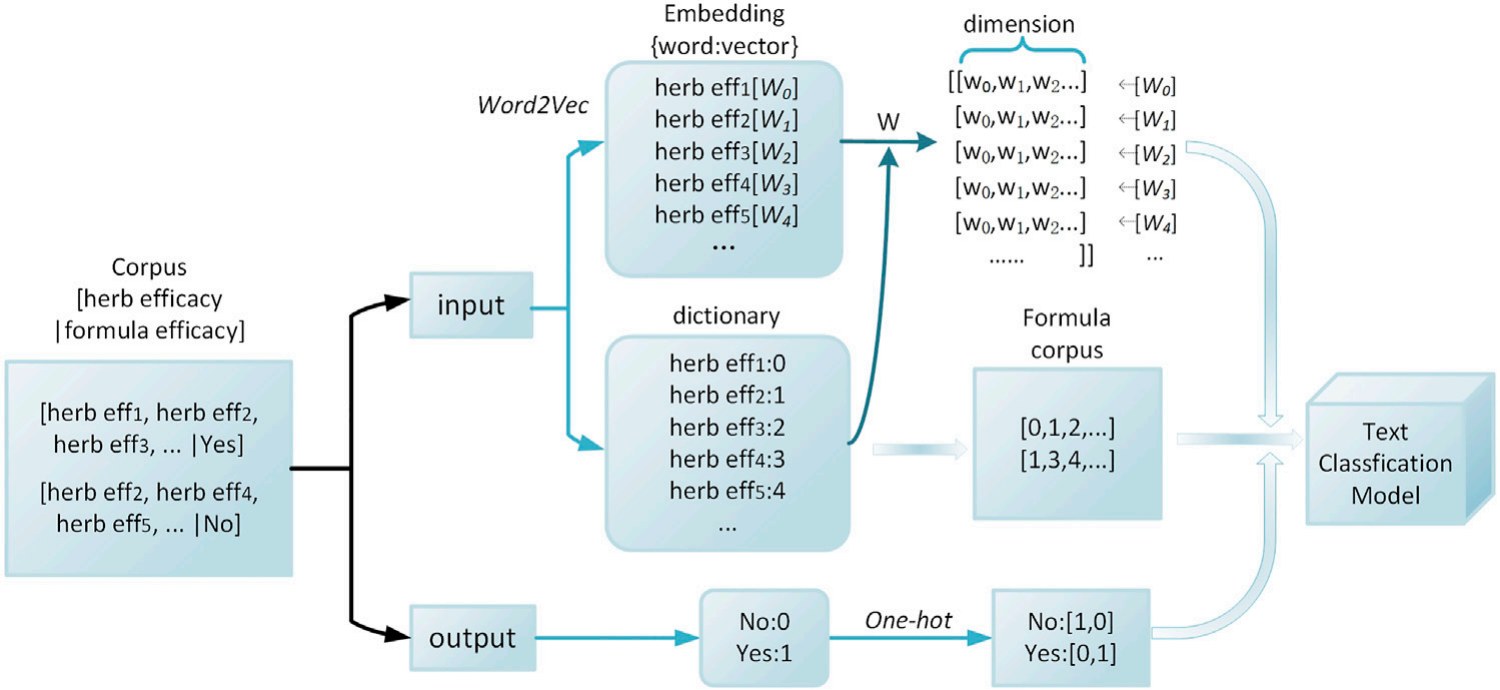
Word embedding layer.

As a formula consists a selection of herbs, formula vector can be a corpus formed by the combination of herb vectors. The herbs can be coded according to their efficacy based on the words as expressed. Word2vec is adopted for herbs while One-hot is adopted for formula efficacy. Figure 2 shows an example of the word embedding layer in classification models. In addition to constructing the herb-vector by the herb efficacy, the herb-vector can also be constructed by the herb name or properties. The method of constructing herb-vector by herb name is the same as above. The method of constructing herb-vector by herb properties is to set a 23-dimensional quantized vector composed of ‘0’ and ‘1’. The 23 descriptions are four characters, five tastes, channel tropism, and toxicity. Dimension ‘1’ indicates that this herb has this feature, while dimension ‘0’ is the opposite.

### 2.3 Deep Learning Models

In this part, we introduce some common DL models for TCM formulae classification, including two basic models: TextCNN and LSTM. Moreover, we present a mixed model consisting of CNN and bidirectional LSTM named TextBLCNN.

#### 2.3.1 Basic Models

##### TextCNN

TextCNN is a variant of CNN. TextCNN uses a k-dimensional vector to represent a word in a sentence (Yoon, 2014). We use a five-dimensional vector to represent the herb efficacy in the formula. Herb-vectors are generally embedded by word2vec. Each row of the two-dimensional matrix corresponds to a herb. Before text input, we must make a dictionary based on herbs contained in the formula. Each herb corresponds to a one-dimensional vector. Then, the formula is input as a two-dimensional matrix into CNN for convolution operation. The network model consists of 200 filters whose window sizes are 2, 3, and 4. To avoid gradient disappearance issues, we use the ReLU function as an activation function. If the eigenmaps are obtained, we pool them according to the maximum value of each convolution value. Max-pool can reduce the parameters of the model and ensure that a fixed-length fully connected layer input is obtained in the output of the variable-length convolutional layer.

Feature extraction is the main function of the convolution layer and pooling layer. It can extract main features from the text sequences of certain lengths through local word order information and further synthesize the main features into advanced features. Convolution and pooling operations make TextCNN omit the feature engineering steps in traditional machine learning.

##### LSTM

RNN is a variable-length neural network, which is widely used in text classification. The RNN model has a short-term memory function, so it is more suitable for processing natural language and other sequence problems (Liu et al., 2016). The traditional RNN model usually has the problem of gradient disappearance and explosion when processing long texts. As an improved RNN network, LSTM can effectively control historical information by adding three gating units and a memory cell (Hochreiter and Schmidhuber, 1997). The internal gating mechanism does not completely discard the hidden state of the previous moment like RNN but selectively preserves the state of the previous moment, which makes it better in processing long sequences of text while solving the problem of gradient disappearance.

LSTM consists of input gate i , forgetting gate f , and output gate h . Input gate i determines how many current network element states $c_{t}$ are passed to the next layer. Forgetting gate f determines how many previous network element states $c_{t-1}$ are retained to the present moment. Output gate h determines how many current unit states are output to the present moment. The equations are shown in Eqs 2.1, 2.2, and .2.3.

$$i_{t}=\sigma \left(W_{i}*\left[h_{t-1},X_{t}\right]+b_{i}\right)$$ (2.1)

$$f_{t}=\sigma \left(W_{f}*\left[h_{t-1},x_{t}\right]\right)+b_{f}$$ (2.2)

$$o_{t}=\sigma \left(W_{o}*\left[h_{t-1},x_{t}\right]+b_{o}\right)$$ (2.3)

$$\tilde{c_{t}}=\tanh\left(W_{c}*\left[h_{t-1},x_{t}\right]+b_{c}\right)$$ (2.4)

$$c_{t}=f_{t}*c_{t-1}+i_{t}*\tilde{c_{t}}$$ (2.5)

$$h_{t}=o_{t}*\tanh\left(c_{t}\right)$$ (2.6)

$i_{t}$ is the input gate, while $f_{t}$ is the forget gate, and $o_{t}$ is the output gate at moment t . $\tilde{c_{t}}$ is the input in the neuron at moment t . $c_{t}$ is the updated value in the neuron at moment t . $h_{t}$ stores the value of the hidden layer at moment t and before. The value of σ is the activation function sigmoid. W and b are the weight and bias terms, respectively, which are updated by the Adam (adaptive moment estimation) optimizer during the training process to adjust the network output.

#### 2.3.2 TextBLCNN Model

We propose the TextBLCNN model which uses a Bi-LSTM layer and a CNN layer to extract data features. It is capable of multifeature levels for high-accuracy target detection and semantic segmentation.

Bi-LSTM is a bidirectional LSTM that extracts bidirectional features of text at the same time to obtain better classification results. Bi-LSTM can capture the two-way semantic dependence from front to back and from back to front through two LSTMs in different directions, thereby effectively combining contextual information.

CNN usually has three parts: the convolution layer, pooling layer, and full connection layer. The convolution layer has convolution kernels of different sizes, which are used for feature extraction. There are multiple pooling methods for the pooling layer, generally max-pooling and average-pooling. Here, we choose max-pooling. The fully connected layer is responsible for connecting all the features and passing the output results to the classifier. Softmax is used for classification, as is shown in Eq. 2.7. At the same time, in order to further improve the model accuracy, dropout is added between the hidden layer and the classification layer to alleviate over-fitting and improve the generalization ability of the model.

$$\bar{y}=soft\max\left(W_{z}*Z+b\right)$$ (2.7)

**FIGURE 3:**
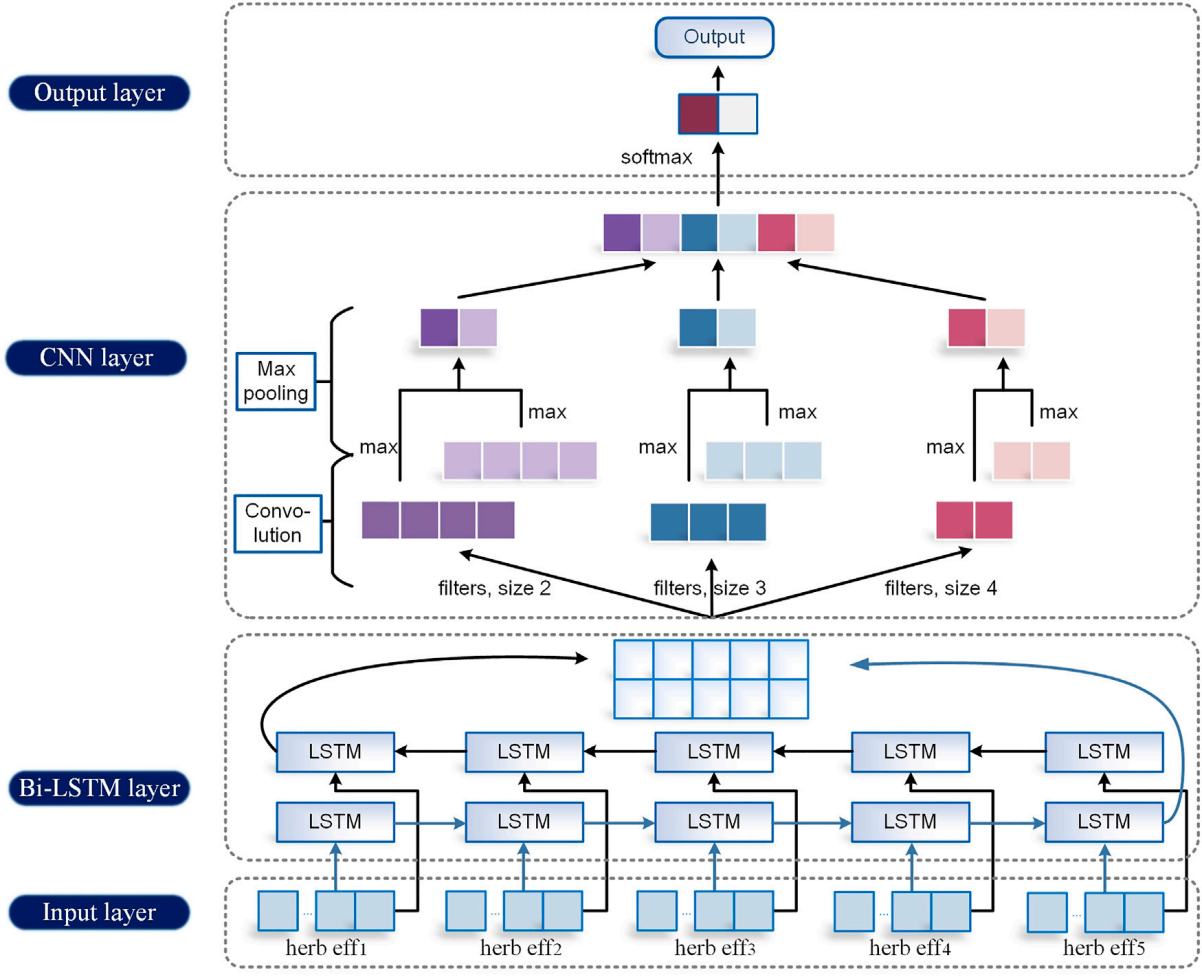
TextBLCNN model.

During training, the batch_size of LSTM is set to 64. The number of neurons is set to 600. The activation function is set to tanh. The number of hidden layers is 1. The size of the convolution kernel in CNN is set to 600*5. The convolution kernel number is set to 200. The step size is [1,1,1,1]. The size of the pooling window is set to 36*1, and no padding is added. The loss function is cross-entropy loss. The learning rate is 0.05. The learning rate attenuation is 0.6. The epoch is set to 20. The overall structure of the model is shown in Figure 3 below.

### 2.4 Over-Sampling Method

Actually, most data are imbalanced. For big data, the problem caused by data imbalance is not obvious, while small data are the opposite. As our data size is relatively small, the imbalanced data will impair the prediction results of trained model towards the category of large samples. Thus, over-sampling is usually used in the data preprocessing stage, so that the proportion of positive and negative classes in the data is balanced by increasing the number of minority samples. However, simply copying the minority samples will increase the possibility of model over-fitting. SMOTE (Synthetic Minority Over-sampling Technique) is an over-sampling technique widely used to synthesize minority samples by randomly selecting similar neighbor samples of a sample for interpolation to generate a new minority sample without repetition. It can overcome the over-fitting problem of random over-sampling to a certain extent.

The basic idea of the SMOTE algorithm is to increase the number of small class samples by interpolation so that the overall data are relatively balanced between positive and negative classes. For any unbalanced data $x_{q}$ , it will randomly select n nearby sample sets. The sample set is expressed as $Z=\left\{z_{1},z_{2},z_{3}\ldots z_{n}\right\}$ . Based on the relationship between the minority samples $x_{q}$ and the nearby sample set Z , the number of minority samples can be increased by interpolation, thereby obtaining the interpolated samples $k_{q}$ . The equation is shown as follows:

$$k_{q}=x_{q}+\xi \left(0,1\right)*\left(z_{i}-x_{q}\right)$$ (2.8)

## 3 Results

To evaluate our models, we use standard indicators for text classification tasks: Acc and F_1_-score. The equations are shown in Eq. 3.1 and Eq. 3.2, TP and FP are the number of positive cases correctly and incorrectly predicted respectively, while TN and FN are the number of negative cases correctly and incorrectly predicted respectively. P is the precision. R is the recall rate.

$$Acc=\;\frac{TP+TN}{TP+TN+FP+FN}$$ (3.1)

$$F_{1}-score=\frac{2*P*R}{P+R}$$ (3.2)

### 3.1 Model Comparison

In order to evaluate the TextBLCNN model, we select several common machine learning classification models for comparisons, such as logistic regression, SVM, LSTM, and TextCNN.

• Logistic regression is a generalized linear regression analysis model.
• SVM is a kind of generalized linear classification based on supervised learning.
• LSTM is a time recurrent neural network, which can solve the long-term dependence problem of general RNN.
• TextCNN is a convolutional neural network specially used for text classification.
• Our TextBLCNN combines Bi-LSTM with TextCNN. The model parameters are shown in Section 2.3.2.

**FIGURE 4:**
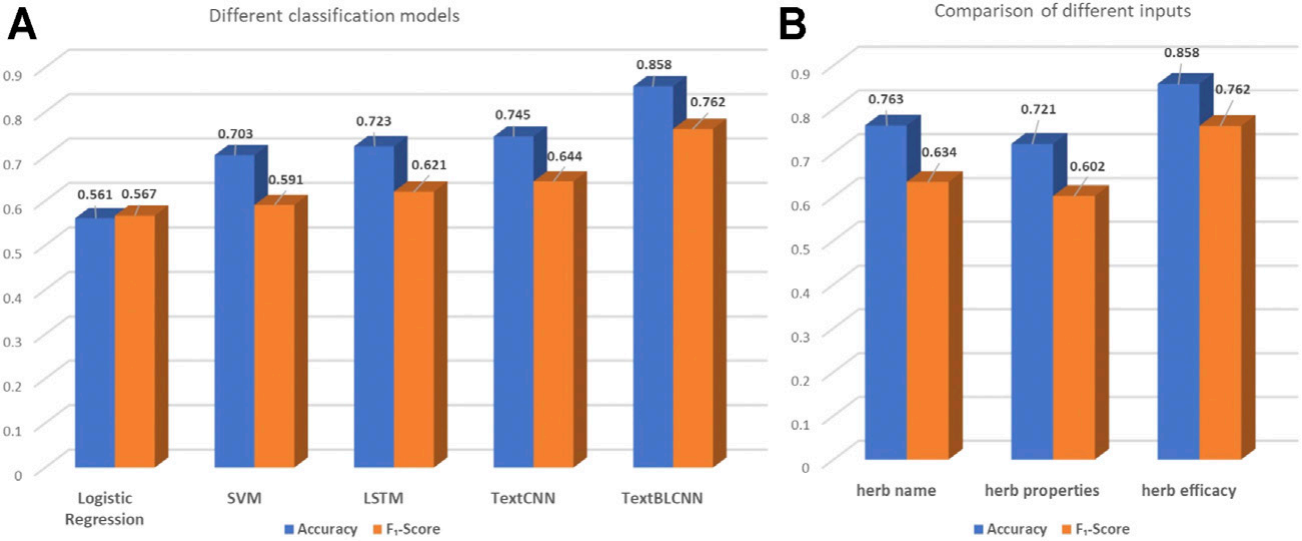
Experimental results.

We select formulae with “regulating blood” efficacy as the positive samples of data that are used for the training of the binary classification model. Because these data have 1,349 positive samples and 1,315 negative samples, these are relatively balanced data. We take 80% of the original data as the training set and 20% of the original data as the test set. The results are shown in Figure 4A. In the case of 2,664 training data, the logistic regression acc is 0.561 and the F_1_-score is 0.567. The SVM acc is 0.703, and the F_1_-score is 0.591. The LSTM acc is 0.723, and the F_1_-score is 0.621. The TextCNN acc is 0.745, and the F_1_-score is 0.644. The TextBLCNN acc is 0.858, and the F_1_-score is 0.762. Compared to other models, TextBLCNN has the highest accuracy rate. The logistic regression model prediction performance is not good. SVM, LSTM, and TextCNN models have relatively accurate predictions. These results show that Bi-LSTM can better capture the two-way features than LSTM, and these features can be better extracted by CNN to achieve higher accuracy in prediction.

### 3.2 Comparison of Different Inputs

For our data, we set three correspondences between formulae and efficacy:

• Predict formula efficacy by the herb name. For each formula, the input of the model is constructed according to different herb names.
• Predict formula efficacy by the herb properties. The 23-dimensional properties of each TCM herb in the formula are superimposed. The 23 descriptions are four characters, five tastes, channel tropism, and toxicity.
• Predict formula efficacy by herb efficacy. For each formula, the herb efficacy is used as a separate word for word embedding pretraining.

We still select the formulae with “regulating blood” efficacy as the data used for the training because of their balance. The TextBLCNN model is used to verify which corresponding relationship can better predict formula efficacy. The TextBLCNN model parameters are shown in Section 2.3.2, and the results are shown in Figure 4B. It can be seen that when the input is the herb efficacy, the model prediction effect is the best. The acc is 0.858, and the F_1_-score is 0.762. When the input is the herb name, the acc is 0.763, and the F_1_-score is 0.634. The herb name does not refer to the characteristic of the herb itself. While the input is the herb properties, the acc is 0.721, and the F_1_-score is 0.602. Therefore, there is a stronger relationship between herb efficacy and formula efficacy. A study once showed that when predicting the incompatibility of herb pairs, differences in efficacy are found to be an important feature, while differences in tastes are not (Zhu et al., 2019). It is also consistent with our experimental results. Therefore, it is inferred that herb efficacy has the strongest correlation with formula efficacy.

### 3.3 Effect of the Over-Sampling Method

In unbalanced data sets, there is a large gap between majority data and minority data, which makes data difficult to classify. In our study, there are a lot of unbalanced data, such as removing phlegm and dispelling pathogenic. There are 875 positive cases and 1,789 negative cases of removing phlegm, while there are 578 positive cases and 2,086 negative cases of dispelling pathogenic. For binary classification, when the proportions of positive and negative samples are similar, the trained model will have better generalization ability. Otherwise, the model prediction results may be biased toward the majority category. Thus, we use the SMOTE algorithm to preprocess part of our data.

Among the 19 categories of formula data preprocessed by the SMOTE algorithm, there are 3,000 positive and negative examples of each type. We use the herb efficacy as the input of the TextBLCNN model to predict the efficacy of each formula. It can be seen from Table 3 that after using the SMOTE algorithm to preprocess formula data, the recall rate of the model has been greatly improved, with an average increase of 47.1%. For each input, the acc does not change much, but the F_1_-score improves greatly. For example, the “carbuncle” model has an acc of 0.925 and an F_1_-score of 0.223. After SMOTE preprocessing, the “carbuncle” model acc is 0.935 and the F_1_-score is 0.938, which shows that the recall rate of the model has been greatly improved.

**TABLE 3:** Comparison of models before and after using SMOTE.

Models formula efficacy	TextBLCNN		S-TextBLCNN	
	Acc	F_1_-score	Acc	F_1_-score
Moistening dryness (治燥)	0.955	0.314	0.933	0.971
Invigoration (补益)	0.779	0.720	0.843	0.838
Regulating blood (理血)	0.858	0.762	0.899	0.812
Dispelling pathogenic (祛风)	0.797	0.409	0.828	0.848
Removing phlegm (祛痰)	0.692	0.671	0.779	0.778
Carbuncle (痈疡)	0.925	0.223	0.935	0.938
Eliminating dampness (祛湿)	0.808	0.294	0.867	0.882
Regulating Qi-flowing (理气)	0.796	0.782	0.844	0.827
Warming (温里)	0.880	0.489	0.939	0.885
Reconciliation (和解)	0.902	0.211	0.908	0.911
Clearing heat (清热)	0.827	0.623	0.861	0.852
Emergency (救急)	0.955	0.195	0.976	0.928
Tranquillization (安神)	0.913	0.214	0.922	0.813
Improving eyesight (明目)	0.943	0.188	0.950	0.972
Purgation (泻下)	0.917	0.199	0.932	0.952
Resuscitating (开窍)	0.850	0.246	0.873	0.899
Resolving food (消食)	0.868	0.277	0.920	0.846
Astringing (固涩)	0.943	0.194	0.962	0.983
Relieve exterior syndrome (解表)	0.932	0.192	0.946	0.975

## 4 Discussion and Conclusion

The scientific connotation of TCM syndrome differentiation and treatment has always been the key direction of the formula study (Wang, 2015). Large amounts of TCM data provide abundant samples for machine learning, DL, and other technologies to explore the scientific connotation of formulae. In recent years, there have been some studies that combine DL with TCM formulae. For example, an OSPF framework with ANN could provide an accurate reference for the TCM formula (Wang et al., 2021). The nonlinear relationship between formulae and symptoms could be established through a neural network (Lin et al., 2018). The result of a DL model (FordNet) for formula recommendation is very similar to those of well-known Chinese medicine doctors (Zhou et al., 2021). NLP and the neural network technology can be combined well to reveal the dialectical relationship between syndrome elements and syndrome differentiation (Wen-Xiang et al., 2019). An adaptive DL model is proposed to aid the dialectic of infectious fever symptoms (Liu et al., 2020). Currently, there is very little research on TCM formulae that uses DL to study the relationship between TCM herb and formula efficacy. This study uses the NLP technology to express TCM herb as herb-vector on the basis of standardized TCM data. It makes herb-vectors of similar efficacy or properties closer in the high-dimensional space. Thereby, it can reflect the correlation of herbs through herb-vector. We use DL to explore inner rules of formula efficacy contained in TCM data. It not only helps to systematically clarify the modern scientific connotation of formula compatibility and provides a basis for TCM clinical syndrome differentiation and treatment but also provides a new way to analyze the basic theory of TCM.

In this paper, we discuss three herb coding methods for formula-vector, namely, herb name, herb properties, and herb efficacy. The formula-vector composed of herb efficacy has the best effect on the classification model because there is a strong relationship between herb efficacy and formula efficacy. An improved DL model S-TextBLCNN is proposed to solve the formula classification problem. The Bi-LSTM layer and the CNN layer are used to extract data features. We study different construction methods and compare the TextBLCNN model with different machine learning and DL models. Experimental results show that our method achieves a higher accuracy rate than the existing classifier method. Aiming at the problem of too low recall rates caused by data imbalance, the SMOTE algorithm is proposed to solve it. After solving the problem of data imbalance, the F_1_-score has been greatly improved by an average of 0.471 in 19 models.

Nevertheless, there are some limitations with this model. For example, as the amount of data increases, the running time cost of the model becomes higher. Therefore, in future research, the big data and distributed framework could be built to reduce the time cost. In addition, formula ingredient dosage is directly associated with efficacy. On this account, we will add the relative dose of TCM herb in the follow-up study when building formula-vector and test the influence of different word-embedding models on the final performance.

## Data Availability

The datasets presented in this study can be found in online repositories. The names of the repository/repositories and accession number(s) can be found in the article/Supplementary Material.
